# Long intergenic non-coding RNA 00473 promotes proliferation and migration of gastric cancer via the miR-16-5p/CCND2 axis and by regulating AQP3

**DOI:** 10.1038/s41419-021-03775-9

**Published:** 2021-05-15

**Authors:** Shuaishuai Zhuo, Miaomiao Sun, Rumeng Bai, Die Lu, Shihao Di, Tianshi Ma, Zigui Zou, Hongxia Li, Zhihong Zhang

**Affiliations:** 1 Department of Pathology, The First Affiliated Hospital of Nanjing Medical University, 300 Guangzhou road, 210029 Nanjing, Jiangsu Province China; 2 Department of Pathology, Zhejiang Provincial People’s Hospital & People’s Hospital of Hangzhou Medical College, 310014 Hangzhou, Zhejiang Province China; 3 Department of Pathology, The First Affiliated Hospital of Soochow University, 899 Pinghai Road, 215000 Suzhou, Jiangsu Province China

**Keywords:** Gastric cancer, Long non-coding RNAs

## Abstract

Gastric cancer (GC) is one of the most common malignancies worldwide, but its molecular mechanisms remain unclear. Increasing evidence indicates that long non-coding RNAs (LncRNAs) play a pivotal role in various cancers recently. Our present study focused on exploring the function of long intergenic non-coding RNA 00473 (LINC00473) in GC. In this study, we found that LINC00473 expression was aberrantly increased in tumor tissues compared with the paired para-cancerous tissues. The expression of high LINC00473 in GC was notably correlated with a higher risk of lymphatic metastasis, a higher incidence of vascular cancer embolus, and advanced TNM stage. Further experiments showed that the overexpression of LINC00473 could promote the proliferation and metastasis of GC cells both in vitro and in vivo. The apoptosis of GC cells increased significantly by the decrease of LINC00473. Mechanistically, LINC00473 could sponge miR-16-5p in the cytoplasm and relieve its suppression of CCND2. Moreover, AQP3 was found to be a significant downstream target gene for LINC00473 through RNA transcriptome sequencing, as demonstrated by qRT-PCR and western blot. Overexpression of LINC00473 can partially reverse the effects of AQP3 decrease on GC proliferation and metastasis. LINC00473 regulated AQP3 expression through CREB was confirmed by western blot. Our research indicates that LINC00473/miR-16-5p/CCND2 axis plays a role in the proliferation of GC and modulates AQP3 to influence GC cell metastasis, making it a potential therapeutic target for GC.

## Introduction

Gastric cancer (GC) is one of the most common malignancies worldwide and the second leading cause of cancer-related death in China^1,2^. In the United States, a significant proportion of patients with GC are diagnosed as late, unfit for surgical treatment, and have an extremely low 5-year overall survival rate^3^. For advanced patients, more prognostic factors and effective biomarkers are needed in addition to conventional therapies. Therefore, elucidating the molecular mechanism of GC is very important.

Long non-coding RNAs (LncRNAs), as a subclass of noncoding RNA of which was transcribed from more than 90% of the genome sequences, can regulate the pathogenesis of various cancers at multiple levels, including GC^4–6^. LncRNAs can influence gene regulation through a number of mechanisms, including epigenetic, transcriptional, and post-transcriptional levels, to influence the progression of cancer. LncRNA-GMAN was significantly upregulated in hepatocellular carcinoma (HCC), which can increase the phosphorylation of eukaryotic translation initiation factor 4B (eIF4B) to promote metastasis and inhibit apoptosis in HCC^7^. Moreover, as a competing endogenous RNA (ceRNA) of microRNA miR-101, lncRNA PTAL can promote the expression of fibronectin 1 (FN1) by sponging miR-101 in ovarian cancer^8^. LncRNAs play an important role in tumorigenesis and development, but the detailed mechanisms that may be useful for diagnosis and treatment of GC remain to be explored.

In the present research, we found long intergenic noncoding RNA 00473 (LINC00473), an 1832 bp ncRNA mapped to chromosome 6q27, was aberrantly upregulated in gastric tumor compared to para-carcinoma tissues. The upregulation of LINC00473 was correlated with a higher risk of lymph node metastasis, a higher incidence of vascular cancer embolus, and an advanced TNM stage. The study also showed that LINC00473 can promote the proliferation and migration of GC cells in vitro and in vivo, and can be achieved by regulating the downstream target gene miR-16-5p and aquaporin 3 (AQP3).

## Results

### The upregulation of LINC00473 and clinicopathological characteristics in GC

**Fig. 1:**
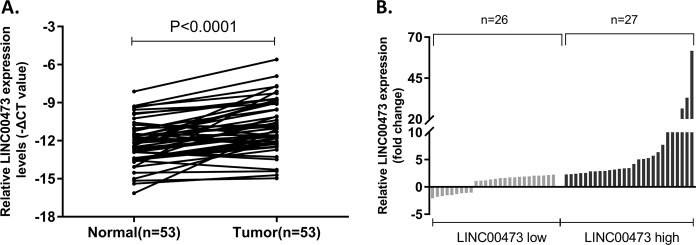
Relative expression of LINC00473 in GC tissues. **A** Relative expression of LINC00473 in GC tissues (*n* = 53) compared with the corresponding non-tumor tissues (*n* = 53) was analyzed by qRT-PCR and normalized against GAPDH expression. The data were analyzed by the paired two-tailed *t* test (*P* < 0.0001). **B** The GC patients were divided into two groups according to the expression of LINC00473. Error bars indicate mean ± standard deviation (SD). *P < 0.05, **P < 0.01.

The expression of LINC00473 was detected in 53 GC tumor tissues compared to corresponding para-carcinoma tissues by qRT-PCR. As a result, LINC00473 expression was aberrantly increased in tumor tissues compared with the paired para-cancerous tissues (Fig. 1A), which was analyzed by the paired two-tailed *t* test (*P* < 0.0001). Next, 53 GC patients were separated into two groups on the basis of the relative expression of LINC00473 (Fig. 1B): relatively high LINC00473 group (*n* = 27, LINC00473 expression ratio ≥ median ratio) and relatively low LINC00473 group (*n* = 26, LINC00473 expression ratio ≤ median ratio) to further explore the clinicopathological characteristics of elevated LINC00473 expression. Table 1 shows the clinicopathological characteristics of the 53 GC patients. High LINC00473 expression in GC was correlated with a higher risk of lymphatic metastasis (*P* = 0.046), a higher incidence of vascular cancer embolus (*P* = 0.009), and advanced TNM stage (*P* = 0.001), but not with age (*P* = 0.560) and gender (*P* = 0.407).

**Table 1:** The correlation between LINC00473 expression and the clinicopathological factors of 53 GC patients.

Characteristics	Number of cases	LINC00473 expression		*P* value
		High (*n* = 27)	Low (*n* = 26)	
*Gender*				0.407
Male	38	18	20	
Female	15	9	6	
*Age*				0.56
≤55	12	7	5	
>55	41	20	21	
*TNM stage*				0.001*
I + II	22	5	17	
III	31	22	9	
*Lymphatic metastasis*				0.046*
Positive	40	24	16	
Negative	13	3	10	
*Vascular cancer embolus*				0.009*
Positive	26	18	8	
Negative	27	9	18	

*P < 0.05 was considered significant.

### GC cell proliferation can be inhibited by LINC00473 downregulation

**Fig. 2:**
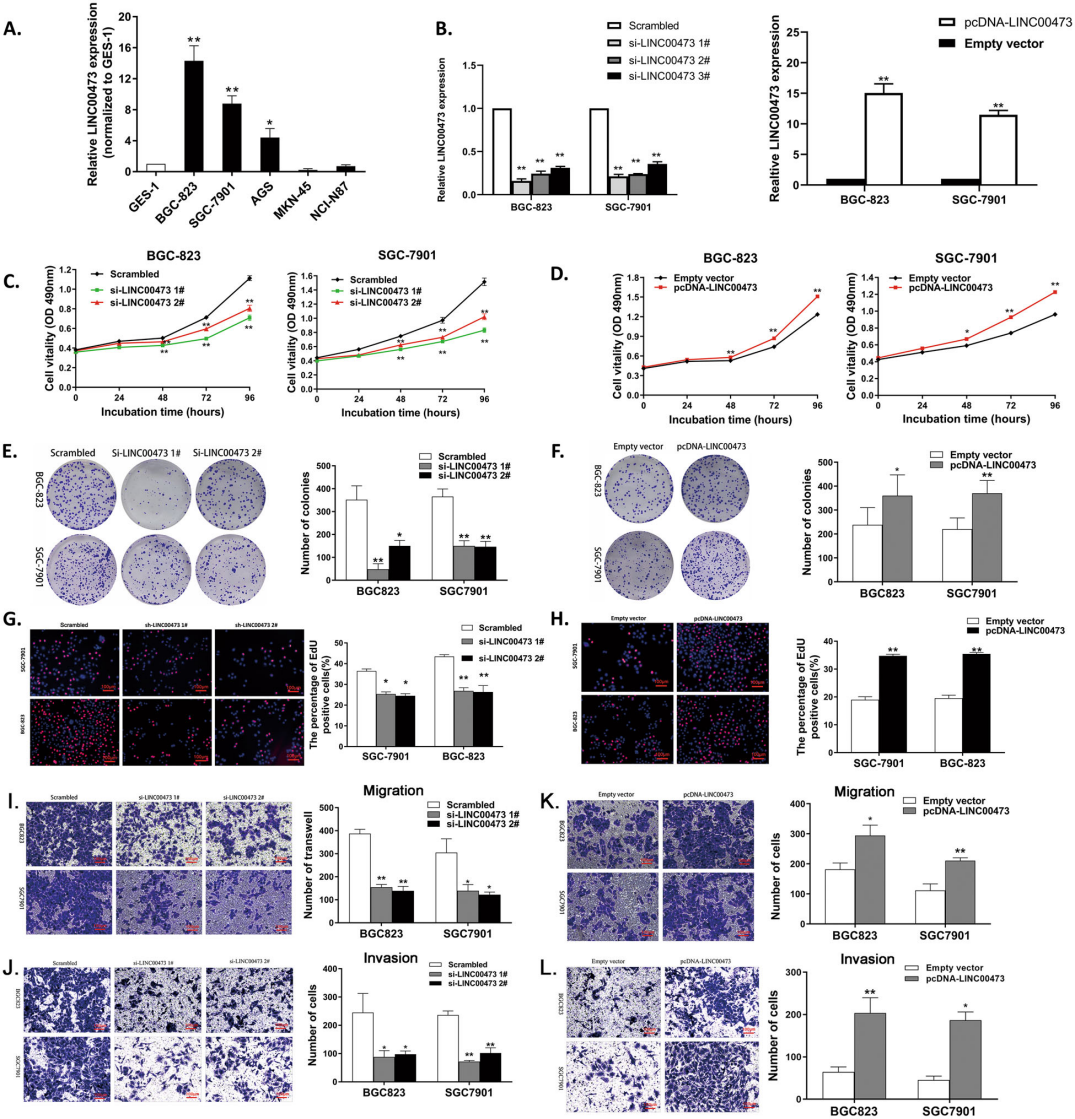
LINC00473 affects gastric cancer cell proliferation and metastasis. **A** LINC00473 expression was detected by qRT-PCR in GC cell lines (BGC823, SGC7901, AGS, MKN-45, and NCI-N87) compared with the normal gastric epithelium cell line (GES-1). **B** Expression of LINC00473 in BGC823 and SGC7901 cells transfected with three siRNAs and pcDNA-LINC00473 were detected by qRT-PCR. **C** and **D** CCK-8 assays were performed to determine the viability of BGC823 and SGC7901 cells transfected with si-LINC00473 or pcDNA-LINC00473. **E** and **F** The proliferation of si-LINC00473 or pcDNA-LINC00473 transfected BGC823 and SGC7901 cells were verified by the colony-formation assays. **G** and **H** EdU immunostaining assays were used to determine the cell proliferation ability of si-LINC00473 or pcDNA-LINC00473 transfected BGC823 and SGC7901 cells. **I** and **J** The migration and invasion abilities of BGC823 and SGC7901 cells transfected with si-LINC00473 or scrambled 24 h were checked by Transwell assays. **K** and **L** Transwell assays were used to determine the cell metastasis of pcDNA-LINC00473 transfected BGC823 and SGC7901 cells. Original magnification, ×100. Scale bar: 100 µm.Values are shown as the mean ± SD based on three independent experiments. *P < 0.05, **P < 0.01.

The expression of LINC00473 in GC cell lines was detected by qRT-PCR analysis, to explore the function in GC cells. The results showed that compared with GES-1, the expression of LINC00473 was upregulated in BGC823, SGC7901, and AGS cell lines, but downregulated in MKN-45 and NCI-N87 cell lines (Fig. 2A). Then, three different small interfering RNAs were designed to silence LINC00473 expression in BGC823 and SGC7901, and qRT-PCR analysis was performed after 48 h of effective transfection. The result showed that there was a higher interference efficiency in si-LINC00473 1# and 2# (Fig. 2B). Therefore, si-LINC00473 1# and 2# were chosen for the follow-up experiments. A pcDNA-LINC00473 expression vector was also devised to induce ectopic LINC00473 overexpression, which was confirmed by the qRT-PCR analysis. The result showed that the expression of LINC00473 in GC cells transfected with pcDNA-LINC00473 was greatly exceeded that of those conducted with an empty vector (Fig. 2B).

Next, the above conditions were used to explore the biological function of LINC00473 in GC cells in vitro. CCK-8 assays revealed that the growth of SGC7901 and BGC823 cells were significantly decreased after impairing LINC00473 expression (Fig. 2C). On the contrary, GC cells conducted with pcDNA-LINC00473 grew better (Fig. 2D). In the colony-formation assays, a decreased clonogenic survival appeared when the expression of LINC00473 was knocked down (Fig. 2E), but the increase occurred after overexpressing ectopic LINC00473 (Fig. 2F). The same conclusion was also confirmed by ethynyl deoxyuridine (EdU) (red)/DAPI (blue) immunostaining (Fig. 2G, H). In summary, these findings suggest that LINC00473 has oncogenic characteristics and can regulate GC cell proliferation.

### Downregulation of LINC00473 decreases GC cell migration and invasion

The invasion and migration of tumor cells are important factors affecting the prognosis of cancer patients. The transwell assay was performed to check whether LINC00473 is related to it. The result showed a decreased number of the GC cells through the artificial matrix membrane conducted with knockdown of LINC00473 (Fig. 2I and J). The migration and invasion ability enhanced with the upregulation of LINC00473 (Fig. 2K and L). These findings suggest that the migration and invasion of GC cells can be regulated by LINC00473.

### Downregulation of LINC00473 induces G1 arrest and apoptosis of GC cells

**Fig. 3:**
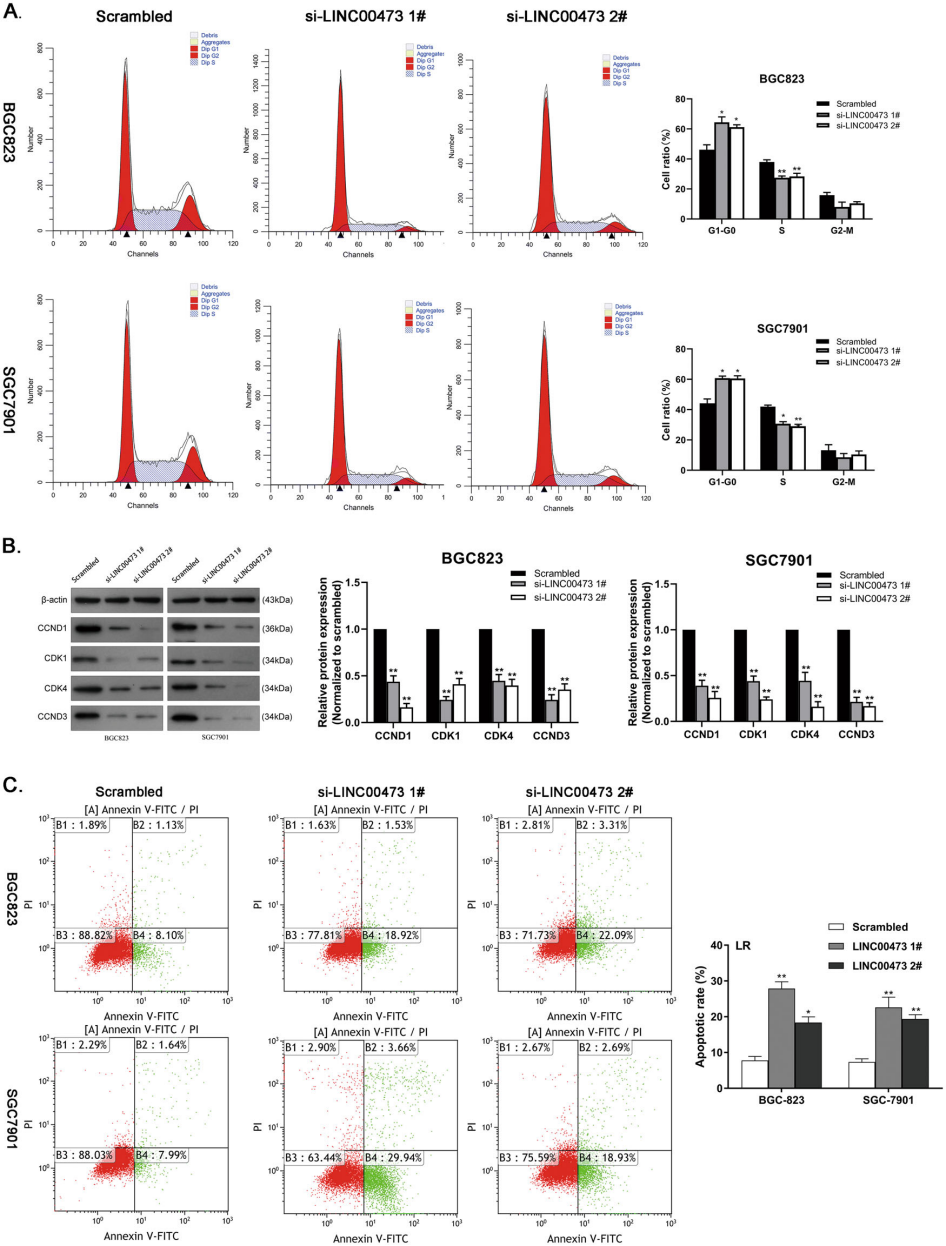
Effects of LINC00473 on the cell cycle and apoptosis. **A** Cell cycle was determined in BGC823 and SGC7901 cells transfected with si-LINC00473. **B** The protein expression of cell cycle-related genes in BGC823 and SGC7901 cells transfected with LINC00473 siRNAs or scrambled were tested by western blot assays. β-actin protein was used as an internal control. **C** Apoptotic rates of BGC823 and SGC7901 cells transfected with LINC00473 siRNAs or scrambled were tested by flow cytometry. LR early apoptotic cells. Values are shown as the mean ± SD based on three independent experiments. *P < 0.05, **P < 0.01.

Next, flow-cytometric analysis was conducted to evaluate the effect of LINC00473 on cell cycle and apoptosis, which is considered to be a key factor in cell growth. BGC823 and SGC7901 cells transfected with si-LINC00473 showed cell-cycle arrest at the G1–G0 phase compared to cells transfected with scrambled control (Fig. 3A). Western blot assays revealed that the protein levels of several cell cycle-related genes (CCND1, CDK1, CDK4, and CCND3) were greatly suppressed in GC cells transfected with LINC00473 siRNAs (Fig. 3B). The increased percentage of apoptotic GC cells was shown after LINC00473 decreased (Fig. 3C). All of the above indicate that LINC00473 participates in the regulation of the cell cycle and apoptosis.

### LINC00473 impacts GC cells growth in vivo

**Fig. 4:**
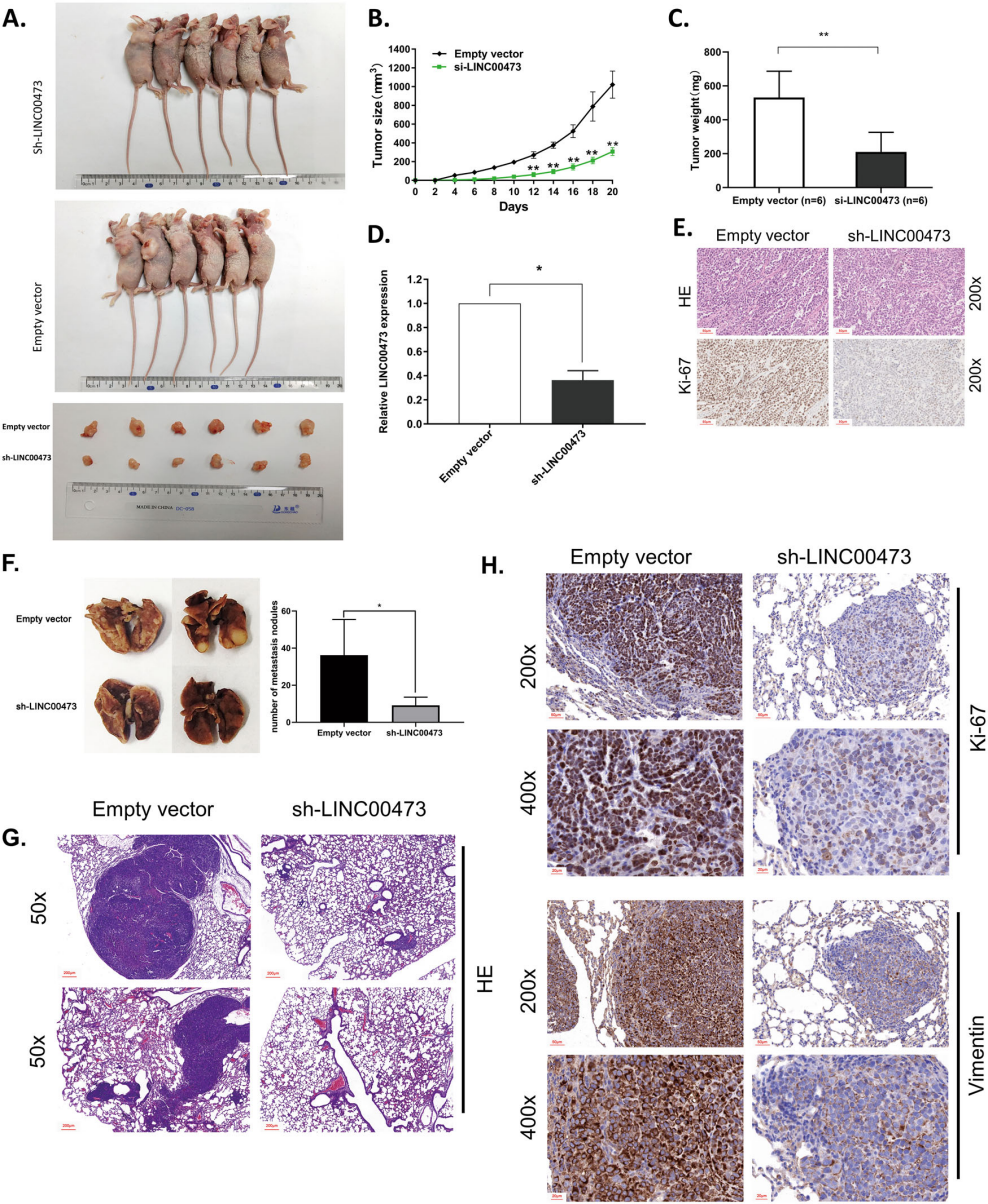
Effects of LINC00473 on GC tumorigenesis and metastasis in vivo. **A** Empty vector or sh-LINC00473 was transfected into BGC823 cells, which were injected into the BALB/c-nude mice (*n* = 6), respectively. **B** Tumor volumes were calculated after injection every 3 days. Points, mean (*n* = 6); bars indicate SD. **C** Tumor weights were represented as means of tumor weights ± SD. **D** QRT-PCR was performed to detect the average expression of LINC00473 in xenograft tumors (*n* = 6). **E** H&E staining and IHC staining with antibodies against ki-67 were used in the tumor sections. Original magnification, ×200. Scale bar: 50 µm. **F** and **G** Lungs from mice in each experimental group transfected with sh-LINC00473 or empty vector, with the numbers of tumor nodules on lung surfaces represented as mean ± SD, are shown. Original magnification, ×50. Scale bar: 200 µm. **H** Lung sections under H&E staining and IHC staining of antibodies against ki-67 and vimentin were showed. Upper: Original magnification, ×200. Scale bar: 50 µm; Lower: Original magnification, ×400. Scale bar: 20 µm. The scale bars were indicated in the figure. *P < 0.05, **P < 0.01.

To explore the influence of LINC00473 on tumorigenesis in vivo, we established the xenograft tumor model. BGC823 cells transfected with sh-LINC00473 or empty vector were inoculated into nude mice. Twenty days later, it was found that xenograft tumors in mice transfected with sh-LINC00473 were smaller than in control mice (Fig. 4A). The tumor growth rate in the sh-LINC00473 group was significantly slower than that of the empty vector group (Fig. 4B). The average tumor weight was obviously lower in the sh-LINC00473 group than that in the empty vector group (Fig. 4C). The qRT-PCR analysis was used to analyze the expression level of LINC00473 in the xenograft tumor tissues. The result showed that the expression level of LINC00473 was much lower in the sh-LINC00473 group compared with the empty vector group (Fig. 4D). Ki-67 staining which can indicate cell proliferation showed a relatively lower intensity in sh-LINC00473 group tumor tissues than control group tissues (Fig. 4E). These results indicate that LINC00473 has the ability to affect tumorigenesis and growth in vivo.

### LINC00473 knockdown impairs GC cells metastasis in vivo

In order to explore whether LINC00473 could affect the metastasis of GC cells in vivo, a model of tail vein metastasis was established. BGC823 cells transfected with sh-LINC00473 or empty vector were injected into the nude mice through the caudal vein. The number of lung metastasis nodules in the sh-LINC00473 group was much lower than that in the control group after 6 weeks (Fig. 4F). The difference between them would be more intuitively on hematoxylin and eosin (H&E) staining of lung sections (Fig. 4G). The ki-67 staining showed that GC cells transfected with sh-LINC00473 had a lower proliferation index. Vimentin, a marker of epithelial–mesenchymal transition (EMT), showed a lower expression in the sh-LINC00473 group than in the other group, suggesting that LINC00473 may affect the metastasis of GC cells via the EMT pathway in vivo (Fig. 4H). The results show that LINC00473 could promote the metastasis of GC cells in vivo.

### LINC00473 acts as a miR-16-5p sponge to regulate the proliferation of GC cells

**Fig. 5:**
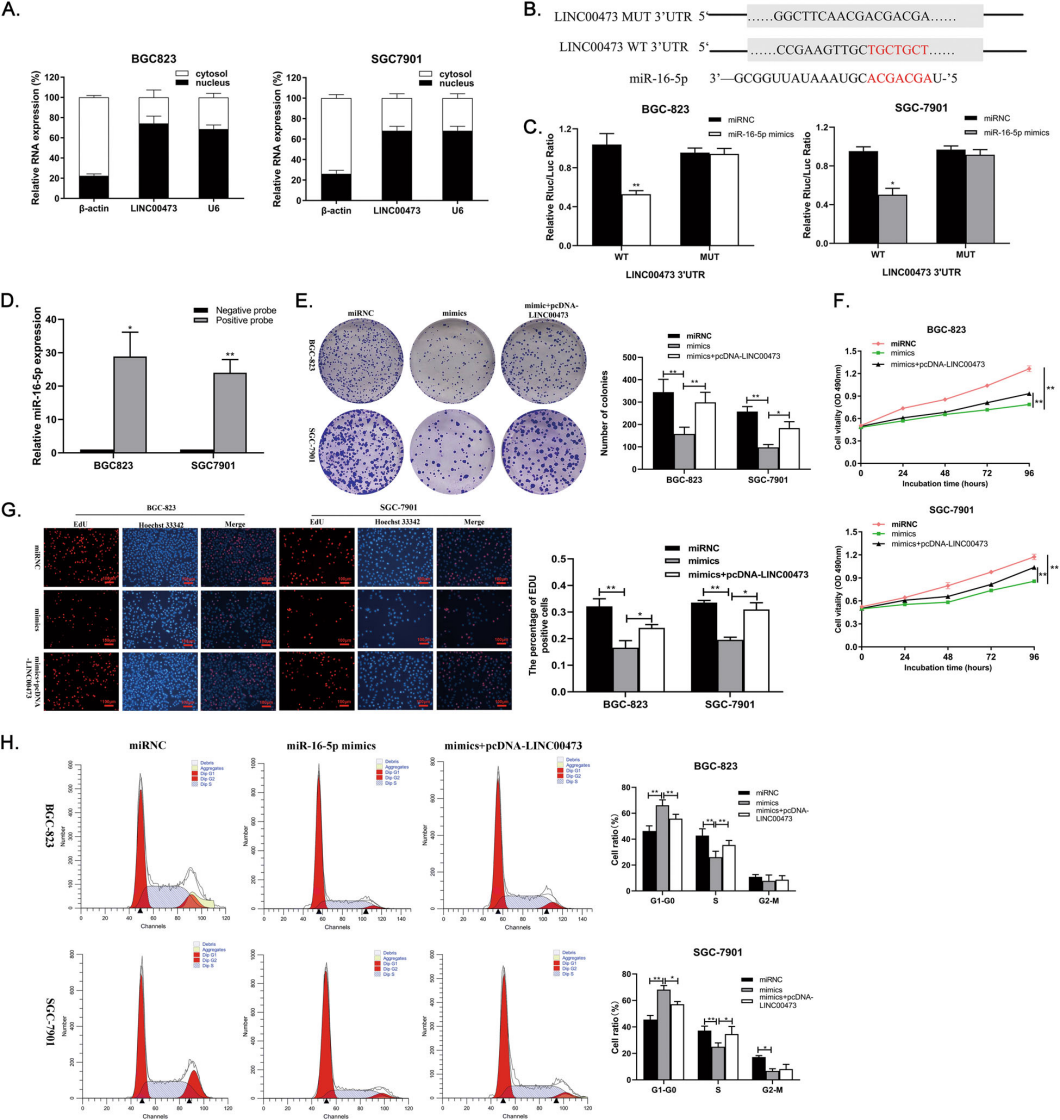
LINC00473 acts as a sponge for miR-16-5p in the cytoplasm. **A** Relative LINC00473 expression levels in nucleus and cytoplasm of BGC823 and SGC7901 cells. Nuclear controls: U6; Cytosolic controls: β-actin. **B** The predicted binding locus between LINC00473 and miR-16-5p. **C** Luciferase activity in BGC823 and SGC7901 cells were co-transfected with miR-16-5p and luciferase reporters containing LINC00473 or mutant transcript. Data are presented as the relative ratio of Renal luciferase activity to firefly luciferase activity. **D** The lysates of BGC823 and SGC7901 cells were incubated with a biotin-coupled positive or negative LINC00473 probe. After pull-down, miR-16-5p was extracted and detected by qRT-PCR. **E**–**G** Colony-formation, CCK-8, and EdU immunostaining assays were used to determine the proliferation for miR-16-5p mimic and pcDNA-LINC00473 co-transfected BGC823 and SGC7901 cells. Original magnification, ×100. Scale bar: 100 µm. **H** Flow cytometry was employed to examine the alteration of BGC823 and SGC7901 cells cycle co-transfected with miR-16-5p mimic and pcDNA-LINC00473. Values are shown as the mean ± SD based on three independent experiments. *P < 0.05, **P < 0.01.

To further investigate the molecular mechanism of LINC00473 regulation in GC cells, the experiment was conducted to determine the subcellular localization of the lncRNA. The results showed that LINC00473 was distributed in both the cytoplasm and nucleus of GC cells (Fig. 5A), which indicated that LINC00473 could play a role in both compartments. There is considerable evidence that lncRNA can play a role in promoting or inhibiting cancer through sponging micro-RNA (miRNA)^9^. ENCORI was employed to calculate the latent miRNA, and it was found that miR-16-5p had a binding site with LINC00473 (Fig. 5B), as confirmed by the Dual-Luciferase Reporter (DLR) assay. MiR-16-5p mimics decreased the luciferase activity of LINC00473-WT in both BGC823 and SGC7901 cells, while LINC00473-MUT treated group showed little change in activity, suggesting that LINC00473 binds specifically to miR-16-5p (Fig. 5C). The data from pull-down assays also showed that the enrichment of miR-16-5p in biotin-coupled positive LINC00473 probe was more than 20 times compared with that in the negative probe group (Fig. 5D). Subsequently, we carried out a rescue assay to further explore the interaction between LINC00473 and miR-16-5p. The decrease of proliferation induced by miR-16-5p mimics could be partially reversed by LINC00473 co-transfection (Fig. 5E, F and G), cell cycle arrest is also reversed as a result (Fig. 5H). Overall, miR-16-5p can repress the proliferation of GC cells, which can be partly reversed by LINC00473 acting as a competitive endogenous RNA (ceRNA).

### MiR-16-5p inhibits the growth of GC cells by targeting the regulation of CCND2

**Fig. 6:**
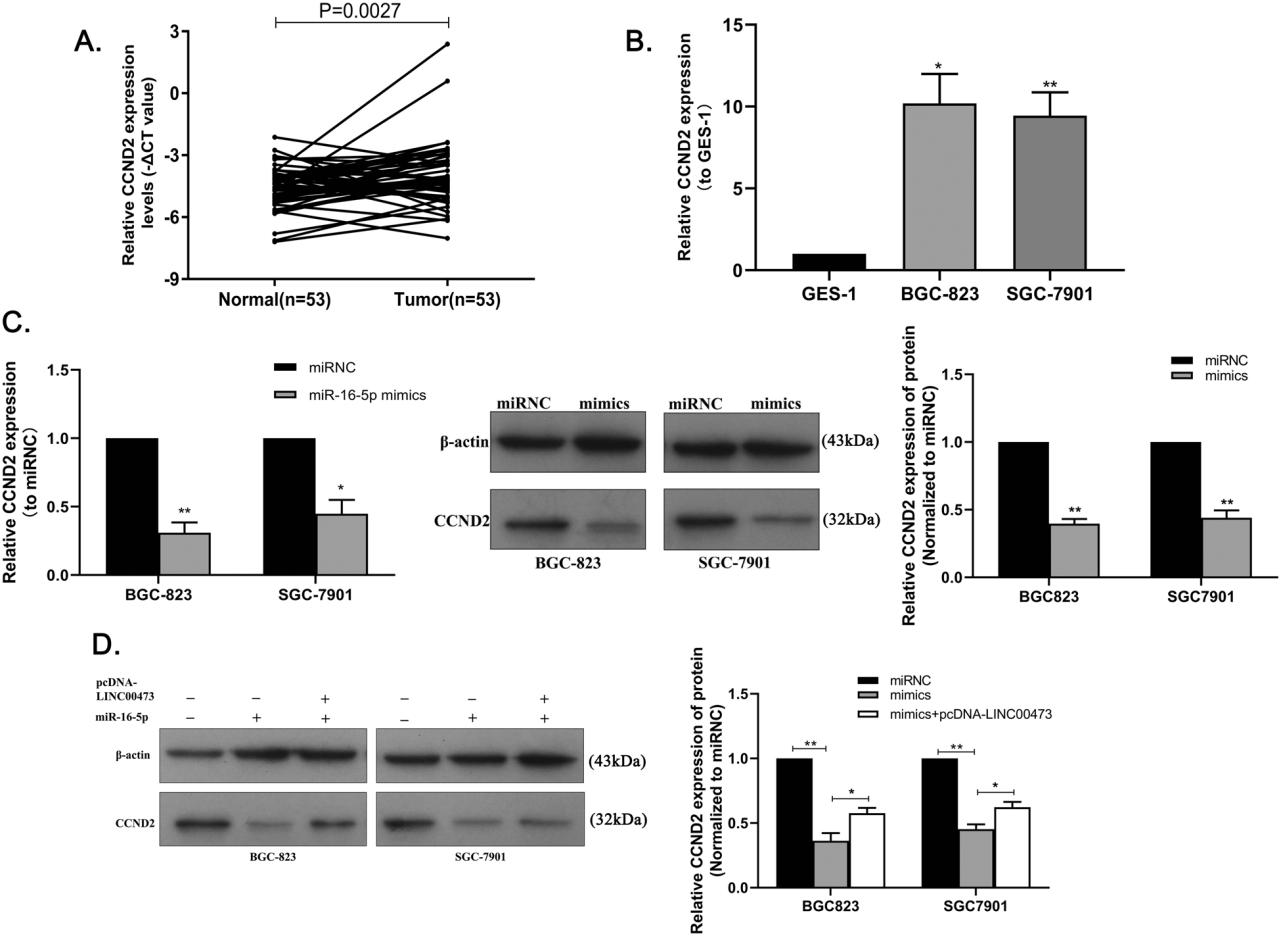
MiR-16-5p sponged by LINC00473 can regulate the expression of CCND2. **A** Relative expression of CCND2 in GC tissues (*n* = 53) compared with the corresponding non-tumor tissues (*n* = 53) was analyzed by qRT-PCR and normalized against GAPDH expression. The data were analyzed by the paired two-tailed *t* test (*P* = 0.0027). **B** The expression of CCND2 in BGC823 and SGC7901 cell lines were detected by qRT-PCR compared with GES-1 expression. **C** Relative expression of CCND2 in BGC823 and SGC7901 cells treated with miR-16-5p mimics both in mRNA and protein level. **D** The alteration of CCND2 protein expression in BGC823 and SGC7901 cells was observed with co-transfected with miR-16-5p mimics and pcDNA-LINC00473. Values are shown as the mean ± SD based on three independent experiments. *P < 0.05, **P < 0.01.

MiR-16-5p-related downstream target genes were identified to further explore the mechanism of miR-16-5p regulating GC proliferation. The result showed that CCND2 has potential binding sites of miR-16-5p analyzed by StarBase v2.0 (Supplementary Fig. 1A), which was validated by DLR assay (Supplementary Fig. 1B). We found that CCND2 was highly expressed in GC tissues than normal gastric tissue analyzed by the paired two-tailed *t* test (*P* = 0.0027) (Fig. 6A), and higher in GC cell lines BGC823 and SGC7901 than in GES-1 (Fig. 6B), which was consistent with LINC00473. The mRNA and protein expression levels of CCND2 in BGC823 and SGC7901 cells transfected with miR-16-5p mimics were suppressed compared with cells transfected with mimics control (Fig. 6C). Overexpression of LINC00473 can partially reverse the effects of miR-16-5p on suppressed CCND2 protein expression level in GC cells (Fig. 6D). These data indicate that CCND2 can act as the target gene of miR-16-5p involved in GC proliferation.

### AQP3 can participate in the function of LINC00473 as an oncogene

**Fig. 7:**
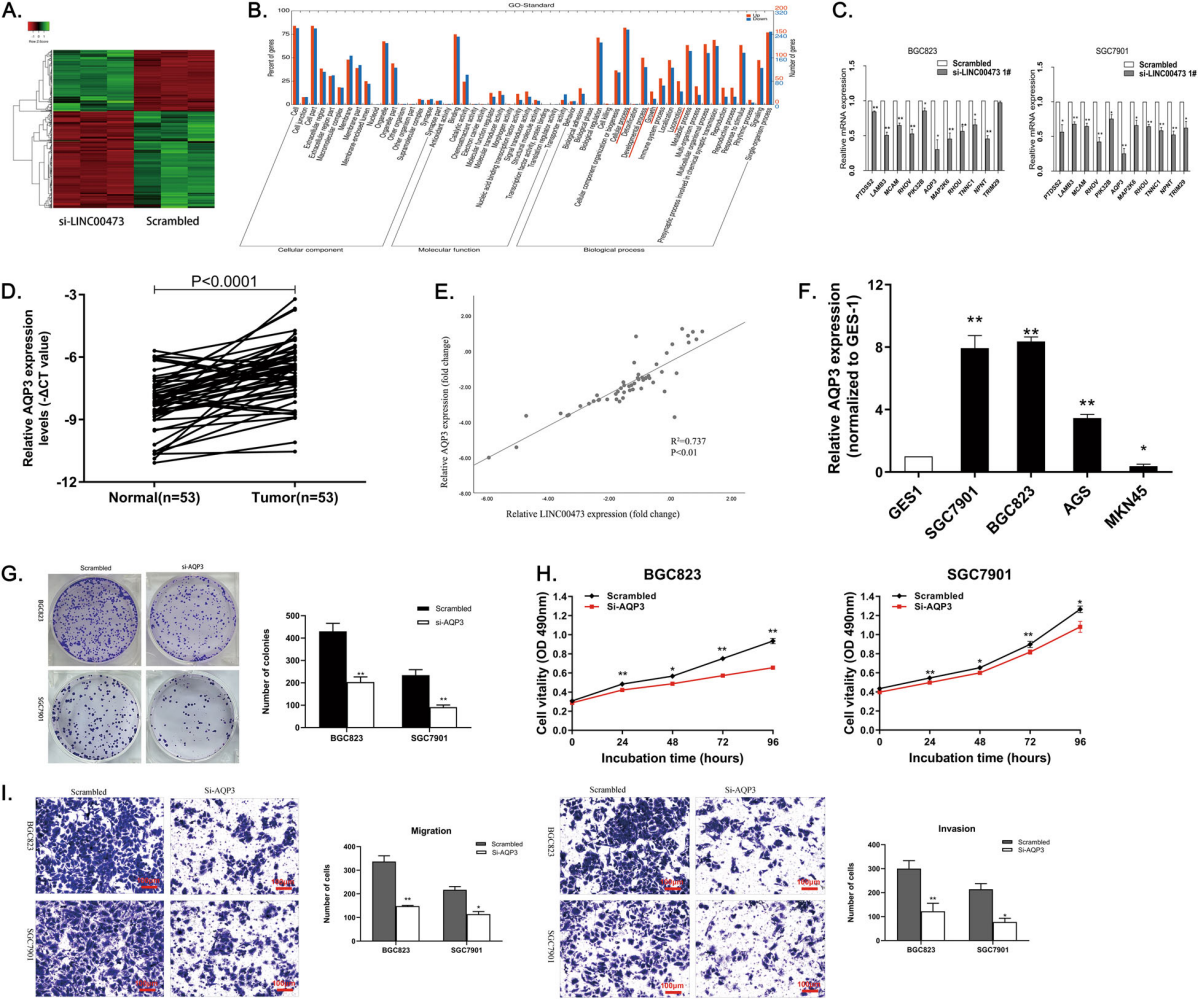
AQP3 is a downstream target gene regulated by LINC00473. **A** and **B** Mean centered, hierarchical clustering of transcripts altered in GC cells treated with scrambled or si-LINC00473, with three repeats. The conspicuous categories of which were concerned in cellular process, developmental process, growth, and locomotion. C QRT-PCR was employed to detect several genes with the most differential expression. **D** Relative expression of AQP3 in GC tissues (*n* = 53) compared with the corresponding non-tumor tissues (*n* = 53) were analyzed by qRT-PCR and normalized against GAPDH expression. The data were analyzed by the paired two-tailed *t* test (*P* < 0.0001). **E** The correlation analysis between LINC00473 and AQP3 expression. **F** The expression of AQP3 in BGC823, SGC7901, AGS and MKN-45 cell lines were detected by qRT-PCR compared with the GES-1. **G**–**I** The proliferation and migration affected by AQP3 were verified by cck-8, colony-formation and transwell assays (24 h after transfection). Original magnification, ×100. Scale bar: 100 µm. Values are shown as the mean ± SD based on three independent experiments. *P < 0.05, **P < 0.01.

Other potential regulatory targets of LINC00473 were detected by RNA transcriptome sequencing. The two groups of BGC823 cells were transfected 48 h with si-LINC00473 1# or scrambled siRNA. The results showed that 574 genes were differentially expressed (fold-change > 2, *P* < 0.05), among which 221 genes were upregulated and 353 genes showing decreased expression in LINC00473 knockdown cells (Fig. 7A). The function of all genes was classified by Gene Ontology (GO)-enriched analysis and significant classifications were found involving cellular process, developmental process, growth, and locomotion (Fig. 7B), which corresponded with our previous experimental results.

In order to find the most suitable target gene of LINC00473 and verify the sequencing results, qRT-PCR was employed to detect several genes with the most differential expression. The result showed that the expression of PTDSS2, LAMB3, MCAM, RHOV, PIK32B, AQP3, MAP2K6, RHOU, TNNC1, and NPNT were suppressed with LINC00473 knockdown. AQP3 was the most downregulated mRNA in GC cells with LINC00473 knockdown (Fig. 7C). The results showed that AQP3 was an important target gene of LINC00473. The correlation between AQP3 and LINC00473 expression level was further detected by qRT-PCR. The expression levels of AQP3 analyzed by the paired two-tailed *t* test (*P* < 0.0001) in 53 GC tissues were notably exceeded compared with paired normal tissues (Fig. 7D), and the expression of AQP3 was correlated positively with LINC00473 expression (Fig. 7E). Compared with the GES-1, the expression of AQP3 was higher in GC cells BGC823, SGC7901, and AGS, but lower in MKN-45 cell lines (Fig. 7F). To verify the role of AQP3 as a tumor promoter, we interfered with the expression of AQP3 in BGC823 and SGC7901 cells. Cck-8 and colony-formation assays presented a lower proliferation of GC cells after AQP3 knockdown (Fig. 7G, H). The migration of GC cells was also inhibited after the knockdown of AQP3 expression proved by transwell assays. (Fig. 7I). These experiments confirm that AQP3 is correlated with the proliferation and migration of GC cells.

Rescue assays were also conducted to verify whether AQP3 can participate in the function of LINC00473 in GC cells. PcDNA-LINC00473 and AQP3 siRNAs were co-transfected to BGC823 and SGC7901 cells. The results showed that the migration of GC cells inhibited by AQP3 siRNAs could be partially reversed by pcDNA-LINC00473 co-transfection (Supplementary Fig. 2A). It is the same as the effect on the proliferation of GC cells (Supplementary Fig. 2B, C). Taken together, these results indicate that AQP3 can partially participate in the role of LINC00473 in the proliferation and migration of GC cells.

### AQP3 can be regulated by LINC00473 through CREB

**Fig. 8:**
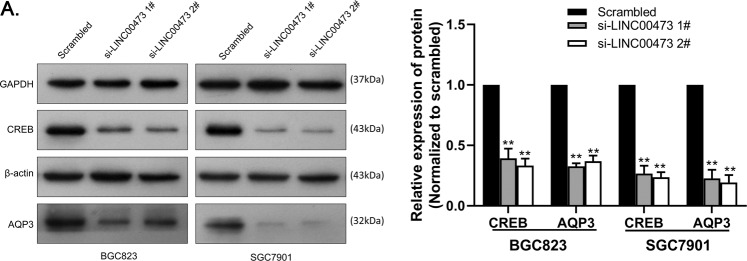
The protein expression of CREB and AQP3. **A** The protein expression of CREB and AQP3 was influenced by LINC00473 knockdown. Values are shown as the mean ± SD based on three independent experiments. *P < 0.05, **P < 0.01.

To further investigate the detailed molecular mechanism of LINC00473 regulating AQP3 in GC cells, we have reviewed the literature. There is evidence that AQP3 can be regulated via the cAMP–PKA pathway, of which CREB is the key molecule^10^. LINC00473 has been shown to function as a facilitator to promote CREB-related transcription in lung cancer^11^. Therefore, we hypothesized that LINC00473 may regulate the function of its downstream target gene AQP3 through CREB, and verified it by western blot. The expression of CREB and AQP3 decreased with the inhibition of LINC00473 in GC cells compared with the control group (Fig. 8A). Taken together, these results suggest that AQP3 is a downstream target gene of LINC00473, which can be regulated through CREB.

## Discussion

The incidence and mortality of GC rank among the top in China and the world. New therapeutic targets are urgently needed to improve patient outcomes, especially in patients with advanced GC^12^. In this paper, we showed LINC00473, highly expressed in GC tissue, could promote the proliferation and metastasis of gastric carcinoma. Mechanistic studies indicate that LINC00473 acts as the ceRNA of miR-16-5p to improve the expression of CCND2 in GC. LINC00473 could regulate AQP3 expression through CREB. These results indicate that LINC00473 functions as an oncogene in GC.

So far, many studies have shown that LINC00473 can promote the progression of different cancers, such as lung cancer, breast cancer, and pancreatic cancer^11,13,14^. The role of LINC00473 in GC has also been reported^15^, but its mechanism remains to be further explored. To explore the function of LINC00473 in GC, the expression level of LINC00473 in GC tissues and adjacent tissues was detected by qRT-PCR. LINC00473 expression was aberrantly increased in 53 tumor tissues compared with the paired para-cancerous tissues. 53 patients were divided into two groups according to the relative expression level of LINC00473, and it was found that the high expression of LINC00473 in GC tissues was correlated with a higher risk of lymphatic metastasis, a higher incidence of vascular cancer embolus, and advanced TNM stage. Among them, the correlation between TNM stage and lymphatic metastasis and the LINC00473 expression was consistent with the previous studies^15^.

The expression of LINC00473 was also detected in GC cell lines, and the results showed that compared with GES-1, the expression of LINC00473 was upregulated in BGC823, SGC7901 and AGS, but downregulated in MKN-45 and NCI-N87. This is different from the previous experimental results, which showed a high expression level in NCI-N87^15^. It was demonstrated that LINC00473 could affect the proliferation of GC cells by functional experiment. Again, this is different from previous research, we speculated that this may be attributed to our use of BGC823 and SGC7901 instead of NCI-N87 and AGS. Moreover, multiple experiments on cell proliferation were employed, rather than cck-8 alone, which may have contributed to the diversity of results. In order to further confirm the role of LINC00473 in the proliferation of GC, a xenograft tumor model in nude mice was established, the results revealed that LINC00473 can promote GC proliferation in vivo. In addition, the effect of LINC00473 on metastasis was also validated, including experiments in vivo.

Due to the different functions of lncRNA located in different subcellular sites^16^, LINC00473 was detected in GC cells and found to be distributed in both cytoplasm and nucleus. We demonstrated that LINC00473 could sponge miR-16-5p in the cytoplasm and relieve its suppression of CCND2. MiR-16-5p is a tumor suppressor for a variety of cancers^17,18^, and CCND2 can promote tumor progression^19,20^. LINC00473 regulates CCND2 expression positively to promote cell cycle progression and thus promote proliferation of GC.

The high expression of AQP3 in GC has been confirmed to be related to migration, proliferation, and adhesion^21^, which was consistent with our experimental results. We confirmed the correlation between LINC00473 and AQP3 and hypothesized that CREB is the key molecule in their interaction. The experimental results showed that LINC00473 altered AQP3 expression by regulating CREB, which affected GC metastasis. Although the feasibility of the mechanism was speculated by consulting previous studies^10,11^ and the correlation between the protein expression was confirmed by WB, the specific action mode of LINC00473 and CREB gene in the nucleus of GC remains to be further studied. One idea is to learn from the mechanism of LINC00473 in lung cancer^11^. The interaction between LINC00473 and NONO was verified by the RNA pull-down assay and western blot. Then, by knocking down the expression of LINC00473, the role of LINC00473 in the recruitment of NONO and CRTC to promote CREB target genes transcription was further verified. Another idea is to use the website to predict the potential binding sites of LINC00473 and CREB promoter, which can be validated by the CHIP assay. Also, more research is needed to find out the mechanism of AQP3 overexpression that accelerates cancer progression.

## Conclusion

We determined the role of the LINC00473/miR-16-5p/CCND2 axis in the proliferation of GC cells and further influenced the metastasis of GC through the regulation of AQP3. Our research suggests LINC00473 a potential therapeutic target for GC.

## Materials and methods

### Cell culture

Four human GC cell lines (BGC823, AGS, MKN-45, NCI-N87) were purchased from the Shanghai Genechem Co., Ltd (Shanghai, China). SGC7901 and the normal human gastric epithelial cell line (GES-1) were purchased from the Shanghai Zhong Qiao Xin Zhou Biotechnology Co., Ltd (Shanghai, China). The cell lines have been authenticated. All GC cell lines were cultured in RPMI-1640 medium (GIBCO-BRL) and GES-1 was cultured in Dulbecco’s modified Eagle medium (DMEM; GIBCO-BRL) in humidified air at 37 °C with 5% carbon dioxide. 10% fetal bovine serum (FBS; Brisbane, Australia) and antibiotics (100 U/ml penicillin and 100 mg/ml streptomycin) (Invitrogen, CA, USA) was added to both media.

### Tissue collection

We collected 53 paired GC and corresponding adjacent nontumorous gastric samples from patients without given local or systemic treatment before surgery. All the patients verified as GC according to histopathological evaluation were operated on at the First Affiliated Hospital of Nanjing Medical University. All tissues collected are immediately frozen and stored in liquid nitrogen. Our study was approved by the Research Ethics Committee of Nanjing Medical University and received informed consent from all patients.

### RNA extraction and qRT-PCR assays

Total RNA was extracted from tissues or cultured cells with TRIZOL reagent (Invitrogen). Real-time PCR analysis was performed using TB Premix Ex Taq (Takara, Dalian, China). The results were standardized by glyceraldehyde 3-phosphate dehydrogenase (GAPDH) expression, and data were analyzed by the comparative cycle threshold (CT) method. The data were presented as the minus delta CT value in the figures. The delta CT value was determined by subtracting the GAPDH CT value from the LINC00473, CCND2, or AQP3 CT value. The exact primers (Invitrogen) are listed in Supplementary Table 1.

### Cell transfection

Three individual LINC00473 siRNAs (si-LINC00473 1#, 2#, and 3#) and scrambled negative control siRNA (si-NC) purchased from Invitrogen were transfected to GC cells together with Lipofectamine 2000 (Invitrogen) according to the manufacturer’s instructions. The Full-length complementary cDNA of LINC00473 was synthesized and cloned into the expression vector pcDNA3.1 (GENEbay, Nanjing, China), and transfected into GC cells by FUGENE transfection reagent (Promega, Wisconsin, USA). The short hairpin LINC00473 (sh-LINC00473) was selected for subcutaneous tumor formation and tail vein metastasis model in nude mice due to its higher transfection efficiency and stable transfection ability. AQP3 siRNAs were synthesized by RiboBio (Guangzhou, China). MicroRNA mimics were also purchased from RiboBio (Guangzhou, China). The detailed sequences are shown in Supplementary Table 1.

### Cell-proliferation assays

Cell Counting Kit-8 (CCK-8, bimake, Houston, USA) was chosen to detect GC cell viability. Cells of different treatments were seeded on 96-well plates, incubated with CCK-8, and observed for changes in absorbance every 24 h. For the colony formation assay, six-well plates were used for appropriate transfected cells, visible cell colonies can be seen 14 days later.

### Transwell assay

For the migration assays, GC cells transfected with si-LINC00473 or pcDNA-LINC00473 were implanted into the Transwell chamber (8 µm pore diameter; Corning Incorporated, New York, USA), and the number of cells passing through the basement membrane was counted 24 h later. For the invasion assays, a diluted Matrigel matrix (300 µg/ml; 100 µl/well; Corning Incorporated) was added into the bottom of the Transwell chamber, followed by implantation of transfected GC cells, and the number of cells passing through the basement membrane was counted 24 h later.

### Flow cytometric analysis

According to the manufacturer’s instructions, GC cells were fixed overnight using 95 percent ethanol and propidium iodide (PI; FcMACS, Nanjing, China), and then counted by flow cytometry to determine the proportion of cells at different cell cycles (G0/G1, S, and G2/M phase). In the detection of apoptosis in GC cells, the proportion of early apoptotic cells in flow cytometry was detected by the Annexin-V-FITC/PI Apoptosis Detection Kit (vazyme, Nanjing, China) 24 h after transfection.

### EdU analysis

YF^®^ 555 Click-iT EdU Imaging Kits (US EVERBRIGHT^®^ INC, Suzhou, China) was used to detect cell proliferation following the manufacturer’s protocol. Cells were cultured in 96-well plates at 5 × 10^3^ cells per well. Then 50 μM EdU labeling medium was added to cells 48 h after transfection, and they were incubated for 2 h at 37 °C under 5% CO_2_. Next, the cultured cells were treated with 4% paraformaldehyde (pH 7.4) for 30 min and then 0.5% Triton X-100 for 20 min at room temperature. Then the samples were stained with anti-EdU working solution and subsequently incubated with 100 μl Hoechst 33342 (5 μg/ml). The percentage of EdU-positive cells was measured under fluorescent microscopy. Five fields of view were randomly selected in each well to calculate the percentage of EdU-positive cells.

### Tumor formation assay and tail vein metastasis in a nude mouse model

Because nude mice have no autoimmunity and immune rejection responses, tumor cells from humans can grow well. The naked skin of nude mice makes it easier to observe tumor formation. The nude mice were chosen as subjects. 4-week-old female BALB/c-nude mice obtained from Charles River (Beijing, China) were cultured under specific pathogen-free conditions and manipulated according to protocols approved by the Shanghai Medical Experimental Animal Care Commission. In the experimental group, a certain number of nude mice of the same species, of the same strain, of the same sex, and of the same age were assigned. Six nude mice in each group were selected for the experiment. A total of 100 μl of suspended cells at a concentration of 1 × 10^8^ cells/ml transfected with sh-LINC00473 or vector control was subcutaneously injected into a single side of the armpit of each mouse. Breeders were blinded to how the nude mice grouped and injected. Tumor volume was measured every 3 days using the equation *V* = 0.5 × *D* × *d*^2^ (*V* represents volume; *D* represents longitudinal diameter; *d* represents latitudinal diameter) after tumor formation. The growth of subcutaneous tumors in each nude mouse was examined after 15 days.

A total of 100 μl suspended GC cells were injected intravenously into the tails of 5-week-old mice at a concentration of 1 × 10^6^ cells/ml. After 15 days of injection, the lungs of the nude mice were removed to observe lung metastasis of GC cells. The study was performed strictly in accordance with the recommendations in the Guide for the Care and Use of Laboratory Animals of the National Institutes of Health. The protocol was approved by the Animal Ethical and Welfare Committee of Nanjing Medical University.

### Subcellular fractionation location

The separation of nuclear and cytosolic fractions was performed using the PARIS Kit (Life Technologies, CA, USA) according to the manufacturer’s instructions.

### DLR assay

MiRNA mimics and double reporter gene carrier or mutant vector were transfected into appropriate GC cells plated in 96-well plates, and incubate for 24 h. Luciferase substrate and stop reagent in Dual-Glo^®^ Luciferase Assay System (Promega Corporation, Wisconsin, USA) were added successively, and fluorescence values were measured using fluorescence illuminometer under the dark conditions.

### RNA pull-down assay

A biotin-coupled LINC00473 probe was specially designed, using an oligonucleotide probe as the control (GenePharma, Shanghai, China). Approximately 1 × 10^7^ GC cells were washed with cold PBS, lysed with cell lysis buffer, and incubated with 50 pmol biotin-labeled probes for 1 h. RNA probes have previously been bound to magnetic beads (Thermo Scientific, USA) for 30 min. The beads were washed three times with wash buffer, and the bound miRNAs were extracted using Trizol reagent and analyzed by qRT-PCR. The sequences of RNA probes are shown in Supplementary Table 1.

### Western blot

Cells protein lysates separated by 10% sodium dodecyl sulfate–polyacrylamide gel electrophoresis (SDS–PAGE) were transferred to 0.22 μm NC membranes (Sigma-Aldrich, USA) and incubated with specific antibodies. The ECL chromogenic substrate was quantified by densitometry (Quantity One software; Bio-Rad, CA, USA). A GAPDH or β-actin antibody (Beyotime, Shanghai, China) was used as a control. CCND1, CDK1, CCND2, CCND3, CDK4 were purchased from Proteintech Group, Inc. (Chicago, USA). CREB was purchased from Cell Signaling Technology, Inc. (CST, Boston, USA). AQP3 was purchased from Abcam (Cambridge, England).

### Statistical analysis

All statistical analyses were performed using SPSS 17.0 software (IBM, SPSS, USA). The significance of differences between groups was estimated by the Student’s *t* test (2 tailed), *χ*^2^ test, or Wilcoxon test, as appropriate. All the data involved in the *t* test were normal distribution. The variation within each group was similar. All data were shown as the mean ± standard errors of the mean based on three independent experiments. *P*-values < 0.05 were recognized as significant. *P < 0.05, **P < 0.01.

## Supplementary information

Supplementary information

Supplementary Table 1

Supplementary Figure legend

Supplementary Figure 1

Supplementary Figure 2
