# Supplementary material for: Long intergenic non-coding RNA 00473 promotes proliferation and migration of gastric cancer via the miR-16-5p/CCND2 axis and by regulating AQP3
Source: Cell Death Dis. 2021 May 15;12(5):496. doi: 10.1038/s41419-021-03775-9 (PMC8124072; doi:10.1038/s41419-021-03775-9)
Supplement: Supplementary file 1 — Supplementary information [file 41419_2021_3775_MOESM1_ESM.docx]

**Supplementary information**

**Supplementary Table 1:** Sequences of primers, siRNAs, shRNA and RNA-pull down probes. xlsx.

**Supplementary Figure 1:** The binding sites between miR-16-5p and CCND2. TIFF.

**Supplementary Figure 2:** Rescue assays of AQP3 and LINC00473. TIFF.

**Supplementary Figure legend:** docx.
