# Supplementary material for: Long intergenic non-coding RNA 00473 promotes proliferation and migration of gastric cancer via the miR-16-5p/CCND2 axis and by regulating AQP3
Source: Cell Death Dis. 2021 May 15;12(5):496. doi: 10.1038/s41419-021-03775-9 (PMC8124072; doi:10.1038/s41419-021-03775-9)
Supplement: Supplementary file 3 — Supplementary Figure legend [file 41419_2021_3775_MOESM3_ESM.docx]

**Supplementary Figure 1 The binding sites between miR-16-5p and CCND2.**

**(**A) The predicted binding locus between miR-16-5p and CCND2. (B) Luciferase activity in BGC823 and SGC7901 cells were co-transfected with miR-16-5p and luciferase reporters containing CCND2 or mutant transcripts. Data are presented as the relative ratio of Renal luciferase activity to firefly luciferase activity. Values are shown as the mean ± SD based on three independent experiments. *P < 0.05, **P < 0.01.

**Supplementary Figure 2 Rescue assays of AQP3 and LINC00473.**

(A) Transwell assays were used to determine the migration of si-AQP3 and pcDNA-LINC00473 co-transfected BGC823 and SGC7901 cells 24 hours later. Original magnification, 100×. Scale bar: 100µm. (B, C) Colony-formation and cck-8 assays were used to determine the cell proliferation of si-AQP3 and pcDNA-LINC00473 co-transfected BGC823 and SGC7901 cells. Values are shown as the mean ± SD based on three independent experiments. *P < 0.05, **P < 0.01.
