# Supplementary figures and images for: Long intergenic non-coding RNA 00473 promotes proliferation and migration of gastric cancer via the miR-16-5p/CCND2 axis and by regulating AQP3
Source: Cell Death Dis. 2021 May 15;12(5):496. doi: 10.1038/s41419-021-03775-9 (PMC8124072; doi:10.1038/s41419-021-03775-9)

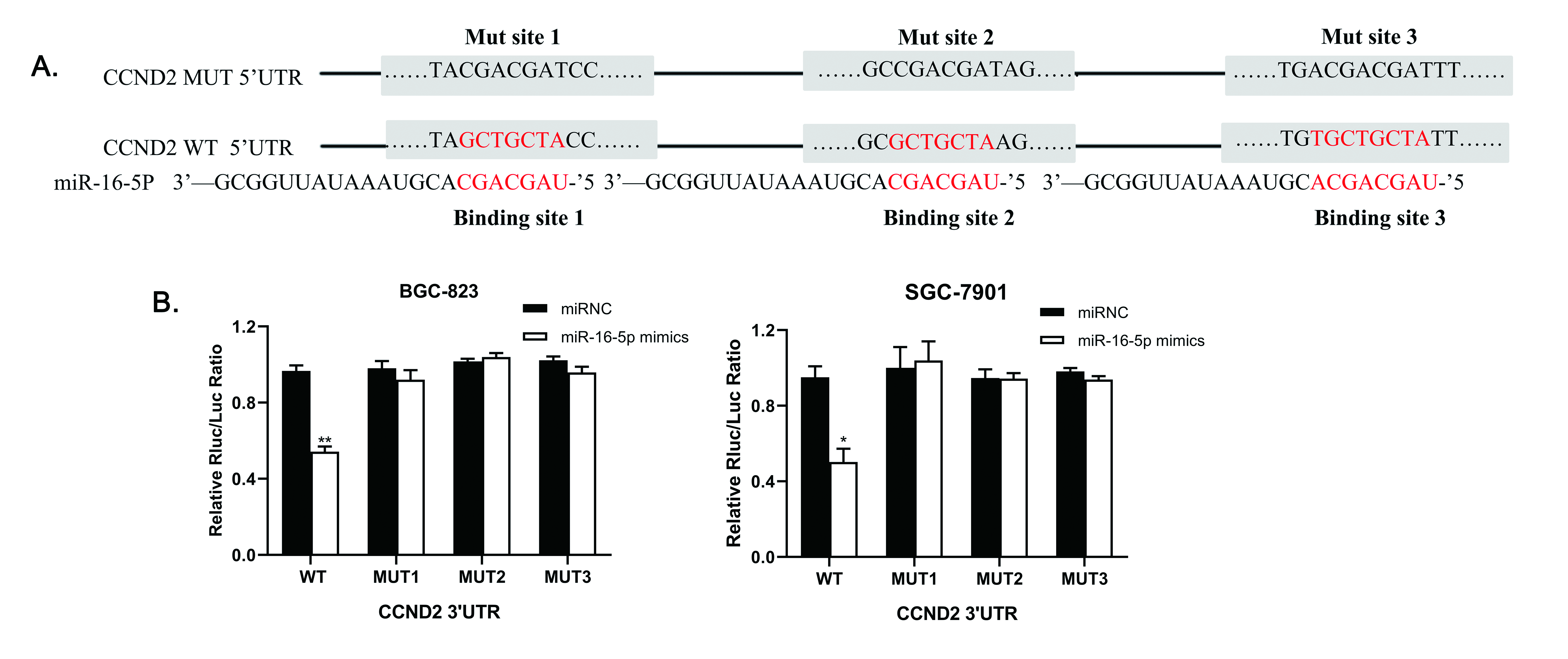

Supplement: Supplementary file 4 — Supplementary Figure 1 [file 41419_2021_3775_MOESM4_ESM.tif]

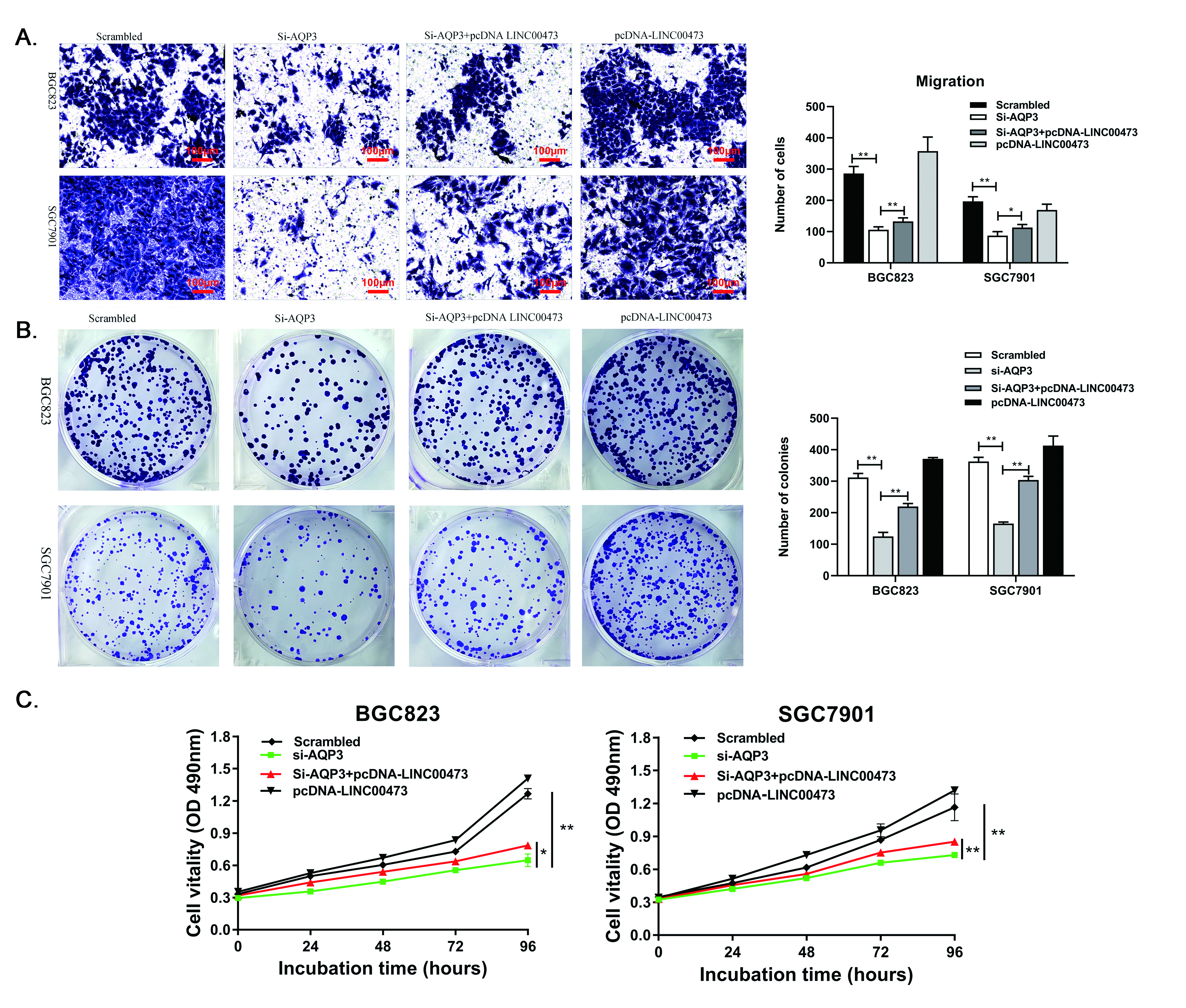

Supplement: Supplementary file 5 — Supplementary Figure 2 [file 41419_2021_3775_MOESM5_ESM.tif]
